# Antibiotic susceptibility in healthcare - Associated infections

**DOI:** 10.6026/973206300210400

**Published:** 2025-03-31

**Authors:** Lalrinpuia B., Seema Rai, Amarpreet Kaur, Gurmeet Kaur

**Affiliations:** 1 Department of Pediatrics, Guru Gobind Singh Medical College, Faridkot - 151203, Punjab India; 2 Department of Pediatrics, Dr. B.R. Ambedkar State Institute of Medical Sciences (AIMS), Ajitgarh - 160055, Punjab, India

**Keywords:** Antibiotics, resistance, intensive care, hospital, infections

## Abstract

The escalating incidence of healthcare infections and the alarming rise of antibiotic resistance underscore the urgent need for a local antibiotic protocol tailored to specific antibiotic sensitivities. Therefore, it is of interest to evaluate the proportion of healthcare-associated infections in the pediatric intensive care units. Hence, we collected 102 samples from patients admitted to the pediatric intensive care units or emergency ward for at least 48 hours with new complaints in the age group of 1 month to 14 years. We identified a total of 102 healthcare-associated infections out of 380 suspected cases, indicating a proportion of 26.84%. Thus, a higher proportion of healthcare-associated infections in pediatric intensive care units are reported.

## Background:

Healthcare-associated infection (HAI) can be defined as "an infection acquired in an acute care setting which was not present or incubating at the time of admission [1]. It was also considered an infection not incubated at admission and arising after 48 hours [2, 3]. In the last decade, significant advancements have been made in the level of care provided to pediatric patients, with the establishment of the first Pediatric Intensive Care Units (PICUs) in 1955 in Sweden [4- 5]. While Indian estimates reported an incidence of 11-23%, western pediatric intensive care units report an incidence of 6-8% [6, 7, 8-9]. Healthcare-associated infections incidence is higher in ICU patients [10]. The most common microorganisms responsible for these infections include *Pseudomonas aeruginosa*, *Acinetobacter baumannii*, *Klebsiella pneumoniae* and *Staphylococcus aureus*, *E.coli* [11]. Bloodstream infections related to central lines are polymicrobial (48%) [12]. the common organisms isolated from bloodstream infection are *Klebsiella pneumoniae*, *coagulase-negative* Staphylococci and *Pseudomonas aeruginosa*, respectively [13]. The common nosocomial pathogens from urinary tract infections are *E.coli*, followed by *Candida albicans* and those infected patients have a five times risk of mortality [14]. All these organisms are responsible for virulent infections and have a high chance of developing drug-resistant infections [15]. According to a study conducted at pediatric intensive care units in Iran and other countries, there is a high incidence of resistance to cephalosporins, fluoroquinolones, carbapenems and aminoglycosides in *Pseudomonas* and *Acinetobacter* infections [16, 17, 18, 19-20]. The intensive care unit has become a high-risk area for hospital-acquired infections [21] and drug-resistant strains because of its large number of special susceptible populations and its particular diagnosis and treatment environment, especially in the pediatric intensive care unit [22, 23, 24, 25, 26, 27- 28]. Therefore, it is of interest to evaluate the proportion of healthcare-associated infections in the pediatric intensive care units.

## Materials & Methods:

The study was conducted in the Pediatric Intensive Care Unit (PICU), Department of Pediatrics, Guru Gobind Singh Medical College & Hospital, Faridkot, from April 2021 to August 2022. The study was a prospective study. All patients admitted to the pediatric intensive care units or in the emergency ward for at least 48 hours and developing new complaints about suspected healthcare-associated infections among the age group of >1 month to ≤14 years. The Exclusion criteria were pediatric intensive care units stay of less than 48 hours, Patients on antibiotics before admission and Refusal of consent. Concerning the previous study of healthcare-associated infection in pediatric intensive care units in Lithuania [23], where 1239 samples were taken, the proportion of healthcare-associated infections was 13.6 %. So, P=0.136, Z=1.96 at 95% CI and with precision (d) = 5%, the sample size (n) becomes 180 using the formula,

n = Z2P (1-P)/d2

The study population (N) will be 200 in the one-and-a-half-year study period. Since the sample size is more than 10% of the population size, the sample size needs to be corrected by using a finite population where *n*_c_ is the corrected sample size. Therefore, the corrected sample size becomes 101.7 and thus, a total of 102 samples were planned to be taken for the study. However, we enrolled 380 patients and consecutive sampling was done. The baseline information was filled in the case record form based on the history obtained from the reliable relatives of the patients. The presenting symptoms, immunization status, developmental history and pre-admission calories and proteins were recorded. A general physical examination and baseline laboratory investigations were sent, including complete blood count, liver function tests, kidney function tests and serum electrolytes. Paediatric sequential organ dysfunction (Paediatric sequential organ dysfunction) score and GCS were noted as soon as they were enrolled in the study. The patients were followed up until they were discharged or died.

## Methodology:

An infection was considered healthcare-associated infections if all elements of a CDC/NHSN site-specific infection criterion were first present together on or after the 3rd day (the day of hospital admission is day 1). Healthcare-associated infections were defined as per CDC/NHSN criteria. The outcomes of the patients were recorded as recovery or mortality. The paediatric sequential organ dysfunction score was used to predict the clinical outcome of the patients [24]. Various clinical samples were collected using standard techniques like blood, urine, pus, tracheal aspirate, catheter tip and body fluids. All samples were labelled and sent to the laboratory with a complete request form as soon as possible.

## Culture and sensitivity testing:

All the clinical specimens from pediatric intensive care units patients received in the Department of Microbiology were inoculated on culture plates (Blood Agar, MacConkey Agar / Cystine Lactose Electrolyte Deficient Agar) and incubated at 37°C for 24-48 hours and direct microscopy was done. The antibiotic susceptibility test of various isolates was performed using the Kirby Bauer Disk Diffusion method per CLSI guidelines [25]. Tops of 4-5 similar-looking colonies were touched with sterile straight wire and inoculated into normal saline and the turbidity was matched with 0.5 McFarland standards. A sterile cotton swab was dipped into the suspension (inoculum), rotated several times and gently pressed onto the inside wall of the tube within 15 minutes of inoculum preparation. The swab was then lawn cultured on Muller Hinton agar plates, the antibiotic disk was applied within 15 minutes of inoculation and the plates were incubated at 37°C for 18-24 hours. The diameters of the zone of inhibition of antibiotics were then measured with the help of the vernier calliper and interpreted as per CLSI criteria. Data were described in terms of range, mean ±standard deviation (± SD), median, frequencies (number of cases) and relative frequencies (percentages) as appropriate. A Kolmogorov-Smirnov test was used to determine whether the data were normally distributed. Comparison of quantitative variables between the study groups was done using the student t-test and Mann-Whitney test for parametric and non-parametric data, respectively. For comparing categorical data, the Chi-square (χ^2^) test was performed and the Fisher exact test was used when the expected frequency was less than 5. A probability value (p-value) less than 0.05 was considered statistically significant. All statistical calculations were done using (Statistical Package for the Social Science) SPSS 21version (SPSS Inc., Chicago, IL, USA) statistical program for Microsoft Windows.

## Results & Discussion:

384 children with suspected healthcare-associated infections of one month to 14 years were enrolled in the study, as shown in Figure 1 - (see PDF). The primary demographic profile of study subjects is shown in Table 1. In the present study, a total of 368 were included in the study, of which a total of 102 healthcare-associated infections were identified. So, the proportion of healthcare-associated infection in the study was 26.84%, as shown in Table 2. Table 3 shows the association of healthcare-associated infections with the different organisms with a p-value of 0.005 (significant). Among the antibiotic-sensitive healthcare-associated infections the majority were *Pseudomonas aeruginosa* and Methicillin-resistant *Staphylococcus aureus* (MRSA) (17.4%) followed by *Acinetobacter baumannii* (15.2%), Methicillin Sensitive Coagulase Negative *Staphylococcus* - MSCONS (13%), *E. coli* (10.9%), Methicillin-resistant *coagulase-negative* staphylococci (MRCONS) (10.9%), *Klebsiella pneumoniae* (6.5%), Meticillin-Sensitive *Staphylococcus aureus* (MSSA) (4.3%) and *Bulkholderia cepacia* complex (4.3%). Among the antibiotic resistant healthcare-associated infections a vast majority of the drug resistant organisms were of *E. coli* (41.1%) followed by Klebsiella pneumonia (17.9%), *Acinetobacter baumannii* (10.7%), *Pseudomonas aeruginosa* (7.1%),5.4% each of MRSA, MRCONS, MSCONS and 1.8% each of Gram negative bacilli and *Bulkholderia cepacia* complex. Among the antibiotic-sensitive healthcare-associated infections in the study, 82.60% of them were discharged, while 17.40% were dead, whereas among the drug-resistant healthcare-associated infections, 80.40% were discharged, whereas 19.60% were dead. The mortality rate was higher among the patients with drug-resistant healthcare-associated infections (p-value 0.804, not significant), as shown in Table 4. Table 5 shows the correlation of antibiotic susceptibility with age, length of stay, Glasgow Coma Scale (GCS) and paediatric sequential organ dysfunction score. The mean age among the antibiotic-sensitive healthcare-associated infections was 3.53 years, while it was 4.92 years for the drug-resistant healthcare-associated infections with a p-value of 0.082, which is not significant. The mean length of stay was slightly longer (18 days) among the antibiotic-sensitive healthcare-associated infections than the antibiotic-resistant healthcare-associated infections (15.75 days), with a p-value of 0.135, which is insignificant. The mean GCS among the antibiotic-sensitive healthcare-associated infections (14.20) was found to be almost the same as the antibiotic-resistant healthcare-associated infections (14.21) with a p-value of 0.960, which is not significant. The mean paediatric sequential organ dysfunction score was slightly higher (2.15) among the antibiotic-sensitive healthcare-associated infections than the antibiotic-resistant healthcare-associated infections (1.75) with a p-value of 0.401, which was not significant.

Among the 368 patients in the study, 102 patients had healthcare-associated infections. The overall proportion of healthcare-associated infections in our study is 26.84 %. In the study of 350 patients by Chowdhury *et al.* [28], 70 had healthcare-associated infections and the overall nosocomial infection rate was 20%. The number of patients was comparable, but our study's proportion was much higher. Most of the international studies by Foglia *et al.* [22] Asembergiene *et al.* [23] and Atici *et al.* [26] show an average incidence rate of 24.58%, which was comparable. In contrast, the Indian studies by Barolia *et al.* [27] show an average incidence rate of 17.74%, which was lower than the international studies. This may be due to differences in the demography and nutritional intake, selection of antibiotics and protocols of escalation and de-escalation among the study population in different studies. The incidence of multidrug-resistant infection (MDRI) was found to be 54.9% in the study, which was very high and may pose severe threats to the management of HAIs and even common infections. *Acinetobacter baumannii* shows 100% sensitivity to Colistin, 87% sensitivity to Meropenem, 83.3% sensitivity to Ciprofloxacin, 66.7% sensitivity to Imipenem, 54.5% sensitivity to Ceftriaxone and 53.8% sensitivity to Piptaz. However, it has a 72.8% resistance to Cefotaxime and 72.7% resistance to Amikacin. This was in contrast to the study by Venmugil *et al.* [29], where they were susceptible to 100% Ciprofloxacin and had a high sensitivity of 67% to Amikacin. *Pseudomonas aeruginosa* was generally sensitive, with 85.7% sensitivity to Colistin, 83.3% sensitivity to Meropenem, 81.8% sensitivity to Ciprofloxacin, 75% sensitivity to Cefepime and Ceftazidime and 70% sensitivity to Amikacin. However, they showed 75% resistance to Ceftriaxone and 50% to Cefotaxime. Barolia *et al.* [27] showed 100% sensitivity to Carbapenems, comparable to our study. Deep *et al.* [30] reported 47% sensitivity each to Amikacin and Ciprofloxacin, which was lesser than the sensitivity seen in our research. Venmugil *et al.* [29] reported a high sensitivity to Amikacin, which was in contrast with our study. *E.coli* was the most common isolate found in the study and showed 100% sensitivity to Colistin, 63% sensitivity to Meropenem, 64.3% sensitivity to Nitrofurantoin and 60% sensitivity to Imipenem. However, it showed a 100% resistance to Cefotaxime and Ceftriaxone, 92.9% resistance to Ciprofloxacin, 82.6% resistance to Piptaz, 75% resistance to Norfloxacin and 50% resistance to Amikacin. Barolia *et al.* [27] reported 50% sensitivity to Carbepenem, comparable to our study. Deep *et al.* [30] reported 55% sensitivity to Amikacin, 80% resistance to Ceftriaxone and 75% resistance to Cefotaxime, which was comparable with our study. Klebsiella Pneumonia showed 88.9% sensitivity to Colistin. Still, it had a resistance of 91.7% to Cefotaxime, 84.6% to Piptaz, 75% to Norfloxacin and Amikacin, 70% to Ciprofloxacin, 66.7% to Meropenem, 60% resistance to Imipenem and 50% resistance to Nitrofurantoin. Deep *et al.* [30] reported a 62.5% resistance to Amikacin, 67.5% resistance to Ciprofloxacin and 67.5% resistance to Cefotaxime, which were comparable. Barolia *et al.* reported 100% sensitivity to Colistin and Carbapenem resistance of 50%, similar to our study. Venmugil *et al.* [29] reported a high sensitivity of *Klebsiella* to Amikacin (83%), Carbapenems (89%) and Ciprofloxacin (67%) which was in contrast to our study. In our study, mortality was higher among healthcare-associated infections patients (18.6%) than among those without healthcare-associated infections (7.9%) (P-value 0.005 significant). This was similar to the study conducted by Asembergiene *et al.* which found that the mortality rate was almost three times higher in patients with healthcare-associated infections than in patients without healthcare-associated infection. Mortality among the antibiotic-resistant healthcare-associated infection (19.60%) was also found (19.60 %%) to be more than the antibiotic-sensitive healthcare-associated infections (17.40%), which was similar to the study conducted by Foglia *et al.* The difference in sensitivity patterns of different centres may be due to the differences in the antibiotic used at other centres and the emergence of a different strain. The strength of study that we conducted antibiotic sensitivity and resistance on all culture sources in pediatric intensive care units it is very strong move towards antibiotic stewardship. Such type of data helps in deciding antibiotic choices depending on the local microbiota. The limitation of the study is we didn't take patient on follow-up. The sample size is less to make guidelines for the region as it is a single center study.

## Conclusion:

The high rate of healthcare-associated infections along with the high rate of multidrug-resistant infections affected mortality and led to prolonged hospitalization.

## Figures and Tables

**Table 1:** The primary demographic of the study subjects

		**HAI group (n=102)**		**No healthcare-associated infections group (n=278)**		**Total**	**Chi- square value**	**p-value**
		**No. of patients**	**%age**	**No. of patients**	**%age**			
	< 1yr	24	23.50%	25	9.00%	49	22.787	0.001
Age group	1-5yrs	46	45.10%	99	35.60%	145		
	>5yrs	32	31.40%	154	55.40%	186		
Male		42	41.20%	112	40.30%	154		
Female		60	58.80%	166	59.70%	226	0.024	0.876
Immunised		87	85.30%	263	94.60%	350	8.895	0.005
Non Immunised		15	14.70%	15	5.40%	30		
Development al		94	92.20%	258	92.80%	352	0.001	0.981
milestones (N)								
Development al		8	7.80%	20	7.20%	28		
milestones (D)								

**Table 2:** Proportion healthcare-associated infections

**Suspected HAI**	**380**
Confirmed HAI	102
Proportion of HAI	26.84%

**Table 3:** Association of antibiotic susceptibility with the different organisms

		**Antibiotic susceptibility**				**Total**	**Chi-square value**	**p-value**
		**Antibiotic sensitive**		**MDRI**				
Organisms	Acinetobacter baumannii	7	15.20%	6	10.70%	13	25.18	0.01
	Pseudomonas aeruginosa	8	17.40%	4	7.10%	12		
	E coli	5	10.90%	23	41.10%	28		
	Klebsiella pneumonia	3	6.50%	10	17.90%	13		
	MRSA	8	17.40%	3	5.40%	11		
	MRCONS	5	10.90%	3	5.40%	8		
	MSSA	2	4.30%	0	0.00%	2		
	Citrobacter freundii	0	0.00%	2	3.60%	2		
	Gram negative bacilli	0	0.00%	1	1.80%	1		
	MSCONS	6	13.00%	3	5.40%	9		
	Burkholderia cepacia complex	2	4.30%	1	1.80%	3		

**Table 4:** Outcome of the study

		**HAI**		**No HAI**		**Total**	**Chi-square value**	**p-value**
		**No. of cases**	**%age**	**No. of cases**	**%age**			
Outcome	Discharge	83	81.40%	256	92.10%	339	8.899	0.005
	Death	19	18.60%	22	7.90%	41		

**Table 5:** Correlation of antibiotic susceptibility with age, length of stay, GCS and paediatric sequential organ dysfunction score

	**Antibiotic sensitive**		**MDRI**		**Z**	**p-value**
	**Mean**	**SD**	**Mean**	**SD**		
Age	3.53	3.6	4.92	4.3	-1.76	0.082
Length of Stay	18	8.7	15.75	6.3	1.508	0.135
GCS	14.2	1.7	14.21	2	-0.05	0.96
PSOFA Score	2.15	2.6	1.75	2.2	0.843	0.401
